# Leisure Time Physical Activities’ Association With Cognition and Dementia: A 19 Years’ Life Course Study

**DOI:** 10.3389/fnagi.2022.906678

**Published:** 2022-06-15

**Authors:** Bente Johnsen, Bjørn Heine Strand, Ieva Martinaityte, Geir Fagerjord Lorem, Henrik Schirmer

**Affiliations:** ^1^Department of Clinical Medicine, UiT The Arctic University of Norway, Tromsø, Norway; ^2^Department of Geriatric Medicine, University Hospital of North Norway, Tromsø, Norway; ^3^Norwegian Institute of Public Health, Oslo, Norway; ^4^Department of Psychology, UiT The Arctic University of Norway, Tromsø, Norway; ^5^Department of Cardiology, Akershus University Hospital, Oslo, Norway; ^6^Institute of Clinical Medicine, University of Oslo, Oslo, Norway

**Keywords:** physical activity, dementia, cognition, cognitive, prevention

## Abstract

**Introduction:**

Cognitive impairment is one of the main disabilities in dementia. Physical activity (PA) has been suggested as protective for dementia. However, the findings are disparate in studies, and the question of whether this is because of reverse causality is still open. We aimed to explore the association of PA with cognition in people who later developed dementia compared to those who did not.

**Method:**

Since 2001, 11,512 (55% women) participants over the age of 50 years had taken at least one cognitive test in the Tromsø Study. Of these, 1,123 (58% women) later developed dementia. The cases were extracted from hospital journals and entered into an endpoint registry. Leisure time PA (LTPA) was self-reported. Multilevel mixed-effects linear regression was used to address whether LTPA was associated with cognition, stratified by those later developing dementia, and dementia-free in a separate analysis.

**Results:**

Leisure time PA was associated with scores in cognitive tests that were 55% (z-score 0.14) higher in those who did not develop dementia. For those in a preclinical phase of dementia, there was no association with LTPA on global cognitive scores. However, in a multifactorial test on processing speed and memory, women had a positive association with processing speed and memory.

**Conclusion:**

Leisure time PA had a positive association with global cognition function only for those who did not develop dementia. In women who were developing dementia, LTPA had a positive association with processing speed and memory, while in men, there were no such associations.

## Introduction

Dementia is a neurodegenerative disease that causes severe cognitive symptoms in a markedly increasing part of the world’s older adults population (Livingston, 2020; Gauthier et al., 2021; GjOra et al., 2021). Physical activity (PA) is suggested as a preventive factor for dementia (Livingston, 2020), through substances such as brain-derived neurotrophic factor (BDNF) (Tari et al., 2019), a neurotrophic and neuroprotective growth factor, which improves brain plasticity and induces neurogenesis and angiogenesis. A decrease in BDNF has been found in people with neurodegenerative diseases, and exercise has been shown to increase BDNF levels in the hippocampus which promotes learning and memory (El Hayek et al., 2019). However, meta-analyses of large observational studies have suggested that the effect of preventing dementia from PA is a reverse causality (Sabia et al., 2017a; Kivimaki et al., 2019). Several trials have aimed to improve cognition or prevent a decline in people with dementia using PA, but the evidence of positive effect on cognition from exercise programmes in people with prevalent dementia is scarce (Cardona et al., 2021). An umbrella review from 2020 found an effect on global cognitive function in patients with dementia, but no effect on attention, executive function, memory, motor speed, or language (Demurtas et al., 2020).

A large Cochrane review in 2015 reported that PA improved the ability to perform activities of daily living, but had no effect on cognition in people with dementia (Forbes et al., 2015). Furthermore, meta-analyses have shown that people with mild cognitive impairment have no significant increase in BDNF with exercise, even though there was a positive trend (Ruiz-Gonzalez et al., 2021). In older adults without dementia, exercise programmes have also failed to show an effect on cognitive outcomes (Sokołowski et al., 2021). However, those with high cardiorespiratory fitness at baseline, or those gaining high cardiorespiratory fitness, have shown improved cognitive abilities over a 5-year period (Sokołowski et al., 2021), suggesting that those without dementia pathology benefits from being physically active, which in turn gives high respiratory fitness over the last 4 decades. LTPA was first decreasing, whereas in the last 20 years, it was increasing for all age groups (Morseth and Hopstock, 2020), while occupational physical activity has been decreasing (Sagelv et al., 2020). These changes have been seen in the population at the same time as cognitive function and grip strength, as a measure of physical capability, and are reported to increase in later born birth cohorts (Strand et al., 2016; Johnsen et al., 2021).

We wanted to see how LTPA was associated with cognitive function in people who later developed dementia compared with those remaining dementia-free for up to 19 years. We hypothesised that a protective effect of PA for the prevention of dementia would result in less cognitive decline in the physically active people who later develop dementia compared to inactive. Alternatively, physical activity could be beneficial by improving cognition, without altering the rate of decline, thereby delaying the onset of dementia.

## Materials and Methods

### The Tromsø Study

The Tromsø Study is an ongoing longitudinal study of the municipality of Tromsø, a city of 76,000 inhabitants in Northern Norway. The first survey was performed in 1974 (Tromsø1), and six more have followed (Tromsø2-7) until 2015/16, approximately 7 years apart (Jacobsen et al., 2012; Eggen et al., 2013). Since 2001, cognitive tests have been included. Representative samples or whole cohorts from the municipality were invited to each survey (Jacobsen et al., 2012; Eggen et al., 2013). They have all consented to the retrieval of medical events or death from hospital records and health registries.

### Study Sample

**FIGURE 1:**
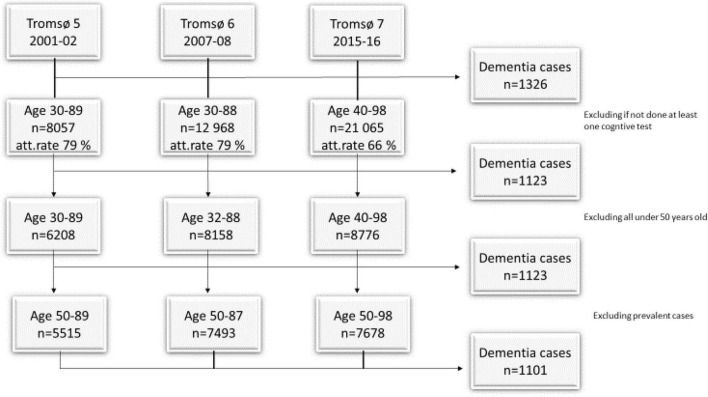
The Tromsø Study 5–7, with survey year, age in years, attendance rate (att. rate), and number of cases (n).

Tromsø5-7 included 27,567 participants, of whom 1,326 later developed dementia. From these, we included 12,710 participants (1,123 later dementia cases) who had performed at least one cognitive test. As all of those who developed dementia were over the age of 50 at participation and because we wanted to see the effect on middle-aged and older adults, we excluded all participants under 50 years old at participation. This left a sample of 11,512 participants with the same frequency of dementia cases. To exclude prevalent dementia cases, those diagnosed with dementia before their first visit (*n* = 21) were excluded. Our final sample consisted of 11,491 participants (55% women). Of these, 1,102 (58% women) later developed dementia. The age range in the first survey was 50–98 years (Figure 1 and Table 1). The maximum follow-up time was 19 years.

**TABLE 1:** Baseline characteristics at first visit in the Tromsø Study.

	Total	Dementia-free	Dementia cases	*P*-value

	*N* = 11491	*N* = 10389	*N* = 1102	
Age at first participation, mean (SD)	62.7 (7.7)	61.8 (7.3)	70.8 (6.4)	<0.001
Age at last participation, mean (SD)	74.4 (9.2)	73.7 (9.2)	81.1 (6.6)	<0.001
Men	45% (5218)	46% (4764)	41% (454)	0.003
Follow up time in years, mean (SD)	10.8 (5.5)	10.9 (5.5)	9.7 (4.9)	<0.001
Physical activity				<0.001
Inactive	29% (3197)	27% (2687)	51% (510)	
Active	55% (5992)	57% (5597)	40% (395)	
Very active	16% (1707)	16% (1619)	9% (88)	
Education				<0.001
7–10 years primary/secondary/technical school	32% (3534)	30% (2996)	53% (538)	
High school diploma (3–4 years)	30% (3296)	30% (2998)	29% (298)	
College/university less than 4 years	18% (2035)	19% (1941)	9% (94)	
College/university 4 or more years	20% (2271)	22% (2184)	9% (87)	
BMI-level in kg/m^2^				<0.001
<18.5	1% (87)	1% (69)	2% (18)	
18.5–25	33% (3803)	33% (3417)	35% (386)	
25–30	45% (5168)	45% (4696)	43% (472)	
30–35	16% (1874)	16% (1687)	17% (187)	
35>	5% (522)	5% (485)	3% (37)	
Hypertension	45% (5072)	43% (4422)	61% (650)	<0.001
Stroke	3% (368)	3% (297)	7% (71)	<0.001
Diabetes	5% (521)	4% (445)	7% (76)	<0.001
Heart attack	6% (644)	5% (531)	11% (113)	<0.001
Smoking				0.07
Yes, now	22% (2460)	21% (2210)	23% (250)	
Yes, previously	44% (5059)	45% (4610)	41% (449)	
Never	34% (3876)	34% (3482)	36% (394)	
Living alone	24% (2759)	23% (2371)	35% (388)	<0.001
Mental status				0.98
No symptoms	35% (3660)	35% (3354)	35% (306)	
Some symptoms	39% (4050)	39% (3703)	39% (347)	
Sub-threshold symptoms	19% (1949)	19% (1786)	19% (163)	
Significant symptoms	7% (762)	7% (699)	7% (63)	

*Description of participants at baseline (first visit), and in addition age at last visit and follow-up time. All values are in percent with n in parentheses if not stated otherwise. P-values obtained by Pearson’s chi-squared test for categorical variables and the t-test for continuous variables.*

### Dementia Register

We constructed a dementia endpoint register for the Tromsø Study (Tromsø1-7). Participants’ national identity number was merged with the diagnosis register at the only hospital in the area, The University Hospital of North Norway. Dementia codes from the International Classification of Diseases-10 (ICD_10) coding Alzheimer’s disease, vascular dementia, Lewy body dementia, Parkinson’s dementia, and other specified and unspecified dementia diagnoses were included (Supplementary Appendix Table 6). The diagnoses were retrieved from the time period of 01.01.1986–31.12.2019. We identified patients whose initial diagnosis was changed over the course of the study period. The register was merged with the Norwegian Cause of Death Registry, but only additional cases were added.

A validation pilot was performed by one geriatric medical doctor (in training). ICD criteria were used to validate the diagnoses in prior medical records. A total of 150 patients, 50 randomly selected from each 5-year time period, from the year 2000 to 2015, were manually checked. Dementia diagnosis had high specificity (99%). The specificity of the subtype was lower (89%); therefore, we did not analyze the dataset for the subtypes of dementia.

The dementia registry was also merged with the ongoing study “The Norwegian registry of persons assessed for cognitive symptoms” (The Norwegian National Centre for Ageing and Health, 2021). Thus, 255 diagnoses for dementia register were validated from the year 2016, finding good consistency in dementia ICD codes.

### Cognitive Tests in the Tromsø Study

A total of four cognitive tests have been performed in Tromsø5, Tromsø6, and Tromsø7. The fifth test (MMSE) was introduced in Tromsø6 and repeated in Tromsø7.

• Word test 1 (WT1), immediate recall, is a test of short-term verbal memory. For this test, 12 nouns were shown on a board and read out loud at 5-s intervals. Points were given for each correctly remembered word within 2 min, 0–12 points.
• Word test 2 (WT2), recognition, is a test of long-term verbal memory, episodic memory, and the ability to use learning strategies. The 12 nouns from WT1 were mixed with 12 new nouns and shown with the same procedure. Participants were asked to identify each word as new or known. One point was given for each correctly identified word, 0–24 points.
• Digit Symbol Coding Test (DSCT), which is a part of the Wechsler Adult Intelligence Scale test battery (Wechsler, 2008), is used to examine psychomotor ability, sustained attention, processing speed, episodic memory, and executive function. It reveals small changes in cognition (Jaeger, 2018). Participants were given a sheet of numbered squares. On top of the sheet, nine symbols were paired with numbers. The subjects were asked to draw the symbol corresponding to the number without skipping a square. One point was given for each correctly identified symbol, 0–96 points.
• Finger Tapping Test (FTT), psychomotor speed. The participants were asked to tap on a plate as many times as they could. After a practice round, they did three rounds of tapping with the non-dominant hand. The points were the mean of the last three rounds, 1–96.
• The Mini-Mental State Examination (MMSE) was introduced in Tromsø6.

At least one cognitive test was performed by 11,491 people over the three surveys: 6,682 did only one, 3,637 did two, and 1,172 did all three. The tests were standardized to *z*-values to allow between-test comparability, and an individual mean score of the performed tests was used as a global cognitive score.

### Measurement of Physical Activity

Leisure time PA was self-reported in two different questionnaires. In all participants under the age of 70 in Tromsø 5, and for all in Tromsø6-7, the Saltin–Grimby Physical Activity Level Scale was used (Grimby et al., 2015). This validated questionnaire had four different categories: inactivity, low, moderate, and vigorous activity (Morseth and Hopstock, 2020). Participants over 70 years old in Tromsø5 were asked two different questions about frequency of light (not sweating or out of breath) or hard LTPA (sweating or out of breath), yielding four categories (Kurtze et al., 2007; Morseth and Hopstock, 2020).

To include all participants in the same LTPA variable, we recoded both questionnaires to a new variable where 0 was inactive, 1 was active, and 2 was very active/athlete (refer to Supplementary Appendix Table 1). Sensitivity tests showed less difference in analysing SGPALS and the three categories of the new variable.

A validation study with participants from Tromsø6 found good correspondence between participants reported physical activity and physical activity objectively measured using an accelerometer (Actigraph LLC) and maximum uptake of O2 [VO_2_(max)]. Correlation between self-reported PA and VO_2_(max) was 0.40, *p* < 0.001 for women and 0.44, *p* < 0.001 for men. Intraclass correlation between accelerometer and self-reported LTPA was 0.62 for women and 0.59 for men (Emaus et al., 2010).

### Covariates

The covariates were chosen from lifestyle factors, including LTPA, education, and comorbidities which are suggested to affect the risk of dementia and cognitive decline (Livingston, 2020). As men and women had significantly different cognitive test scores in a previous study (Johnsen et al., 2021), we stratified the analyses on sex. Mediating factors included blood pressure, diabetes, hyperlipidemia, stroke, heart attack, smoking, mental status, and body mass index (BMI).

Questionnaires were given, and anthropometric measurements were collected during each survey (Eggen et al., 2013). Covariates were used from the same survey as the cognitive tests were done. Education was dichotomized into high and low education, where high education was university or college, and low education was primary education or high school as the highest degree. Hypertension was defined as systolic blood pressure >140 mmHg and/or diastolic blood pressure >90 mmHg and/or use of antihypertensive drugs. Diabetes was a self-reported “yes/no” or the use of anti-diabetic drugs. Hyperlipidemia was defined as total serum cholesterol >5 mmol/l or high-density lipoprotein < 1.0 mmol/l for men; and <1.2 mmol/l for women, or low-density lipoprotein >3 mmol/l, or use of lipid-lowering drugs. Previous strokes and previous heart attacks were self-reported “yes/no.” Smoking was self-reported as never, previous, or current. To assess mental status, we used the Hopkins Symptom Checklist-10 (HCSL-10), a questionnaire designed to measure physiological distress, anxiety, and depression (Strand et al., 2003). BMI was measured and categorized as underweight (BMI < 18.5 kg/m^2^), normal weight (BMI = 18.5–25 kg/m^2^), overweight (BMI = 25–30 kg/m^2^), obese (BMI 30–35 kg/m^2^), and severely obese (BMI > 35 kg/m^2^).

### Statistical Analysis

To visualize how education and physical activity were associated with cognitive scores, plots were constructed by calculating predictions for global cognitive function from a linear regression of global cognitive function on age. To measure the effect size between the groups that were dementia-free and participants who later developed dementia, we used Hedge’s g for LTPA and the global cognitive test score.

To investigate the association between LTPA and cognitive abilities in those who later developed dementia compared to dementia-free, considering the repeated-measures design, multilevel mixed-effects linear regression was used. Models were fitted using the likelihood ratio tests. The time variable was calculated as time from participation in the Tromsø Study to dementia diagnosis, death, or study exit (31.12.2019), whichever came first.

Moderators were added successively, always including the independent variables PA and age in the model.

We first made four models see how different covariates affected the β-coefficients for activity, with a global cognitive score as the outcome. The models were as follows: Model 1: adjusted for age and time, Model 2: Model 1 + education, Model 3: full model, including Model 2, comorbidity, and lifestyle factors. To see whether different cognitive areas, as described under cognitive tests, were affected differently by LTPA, we also ran Model 2 on all cognitive tests separately. Interaction was tested by including the interaction term with age and PA, time and LTPA, sex and LTPA, and education and sex.

Sensitivity testing was done by excluding those who scored low on the cognitive tests to see whether possible prevalent dementia cases caused a lack of association between LTPA and cognitive test scores in those who later developed dementia. Possible prevalent cases were excluded. They were identified by an MMS score < 20 and a test score of 0 on any of the tests (*n* = 489). We also ran sensitivity tests, removing the lower 2.5 percentiles of scores, with no difference in significance and only small changes in beta values. However, n of dementia cases dropped to 897, as all observations for the participant are dropped.

All analyses were performed using Stata 17.0 MP.

## Results

### Description

Mean age at first participation in the Tromsø Study was 61.8 for the dementia-free, and 70.8 for those who later developed dementia (Table 1). Mean age at first dementia diagnosis was 81.6 (CI 81.0–82.1) years for women and 80⋅5 (CI 79⋅9–80⋅1) years for men. Participants who later developed dementia were older at first survey, were less educated, and were less physically active. They were more likely to be hypertensive, have diabetes, report previous heart attack and stroke, and were more frequently living alone, but had the same level of anxiety and depression (Table 1). The Hedge’s g measure for effect size between the groups of dementia/dementia-free was 1.07 for global cognitive function and 0.43 for LTPA. We found 2,307 individuals who had received one or more of these diagnoses in the full registry, and of these, 1,123 had previously performed at least one cognitive test in the Tromsø Study. A total of 568 patients with dementia diagnosis changed subtype of dementia from first to last visit.

**FIGURE 2:**
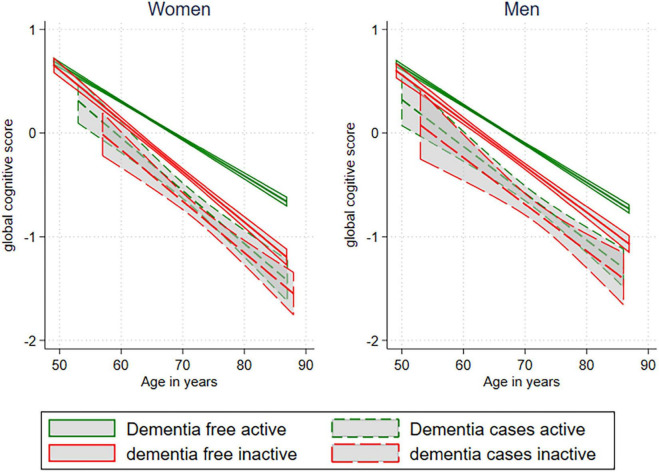
Linear fit prediction plot for global cognitive score, stratified in activity, dementia, and sex.

Dementia-free participants scored higher on cognitive tests than participants who later developed dementia, and women scored on average better than men (Figure 2). The association was tested in regression models, including sex. Women out-tested men in Word test 1, Word test 2, the Digit Symbol Coding Test, and Mini-Mental State Examination, whereas men tested better than women in the Finger Tapping Test, all at *p* < 0.001.

Active dementia-free participants had a better global score on cognitive tests than inactive dementia-free participants did. For those who later developed dementia, being active did not improve their global cognitive test scores, even though there seemed to be a non-significant level difference for the active and inactive in this group as well. Inactive dementia-free women had a steeper decline in cognitive test scores with age compared to inactive, dementia-free men (p-interaction = 0.001). This interaction remained significant when adjusted for education. Inactive dementia-free participants of both sexes also had a steeper decline with age compared to active dementia-free participants (*p* < 0.001) (Figure 2). However, women still outperformed men (*p* < 0.001) if they remained dementia-free. There was no significant effect of sex on those who later developed dementia.

**FIGURE 3:**
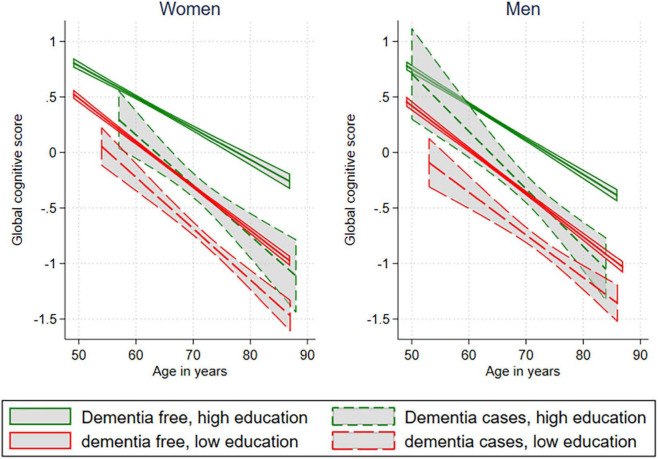
A linear fit prediction plot for global cognitive score on age with a 95% confidence interval of the prediction, stratified on sex and education level. Only adjusted for age.

People of both sexes with high education had higher cognitive scores (Figure 3), compared to those with lower education levels. Participants in the preclinical phase of dementia had lower cognitive scores before the onset of dementia compared to dementia-free subjects with similar education. People with high education prior to dementia onset had similar cognitive test scores as dementia-free with low education at the same age. Men had a steeper decline in cognition if they were higher educated, and they developed dementia later than women in the same group. However, this slope was not statistically significant (p-interaction = 0.623). There was no overall significant interaction between education and activity level with cognitive tests as the outcome, but when tested separately, there was an interaction between education and activity in dementia-free women only.

Physically active participants scored better than those who were inactive on cognitive tests if they were dementia-free (Table 2). However, if they were developing dementia, higher activity levels were not associated with increased cognitive scores, except for short-term memory. Not adjusted for education, however, the difference in short-term memory was significant for very active women developing dementia. This association was no longer significant when education was included in the model. The positive association between LTPA and global cognitive score in dementia-free participants remained after adjusting for all covariates. When tested separately, adjusted for age, time, and education (Table 3), the results persisted, except for the tests Word test 1 and Digit Symbol Coding Test. For dementia-free women, the results on short-term memory (Word test 1) were solid (*p* < 0.01) before adjusting for education, but they were non-significant after adjusting for this covariate. For the Digit Symbol Coding Test, the association remained statistically significant after adjusting for education for both groups, even those who later developed dementia. This relationship was present for both active and very active women, (respectively, *p* = 0.003/0.002) and for very active men (*p* = 0.02). The effect also persisted in the sensitivity tests when those with very low scores were excluded. A positive association between LTPA and the cognitive score was not present in any other tests in those who later developed dementia.

**TABLE 2:** Effect of PA on global cognitive score, mixed effects model.

	Women				Men			
*Z*-values	Dementia-free		Dementia cases		Dementia-free		Dementia cases	
Global CF	β	CI 95%	β	CI 95%	β	CI 95%	β	CI 95%
**Model 1**								
Inactive	Ref	(-)	Ref	(-)	Ref	(-)	Ref	(-)
Active	0.15**	(0.0.12–0.18)	0.04	(−0.07–0.15)	0.12**	(0.09–0.16)	0.03	(−0.09–0.14)
Very active	0.18**	(0.13–0.22)	0.26*	(0.06–0.46)	0.15**	(0.11–0.20)	0.09	(−0.07–0.25)
ICC	0.0.536	⋅⋅	0.422	⋅⋅	0.528	⋅⋅	0.480	⋅⋅
**Model 2**								
Inactive	Ref	(-)	Ref	(-)	Ref	(-)	Ref	(-)
Active	0.12**	(0.08–0.15)	−0.01	(−0.12–0.10)	0.10**	(0.07–0.13)	0.02	(−0.10–0.13)
Very active	0.11**	(0.07–0.16)	0.17	(−0.03–0.37)	0.11**	(0.07–0.15)	0.05	(−0.11–0.22)
ICC	0.495	⋅⋅	0.379	⋅⋅	0.464	⋅⋅	0.453	⋅⋅
**Model 3**								
Inactive	Ref	(-)	Ref	(-)	Ref	(-)	Ref	(-)
Active	0.09**	(0.05–0.12)	−0.05	(−0.17–0.06)	0.08**	(0.04–0.11)	0.02	(−0.10–0.14)
Very active	0.08**	(0.03–0.13)	0.14	(−0.08–0.36)	0.07**	(0.03–0.12)	0.02	(−0.15–0.18)
ICC	0.482		0.328		0.446		0.522	

Multiple mixed linear regression with nested id and global cognitive test score outcome. Model 1: adjusted for time and age, Model 2: Model 1 + education, Model 3: Model 2 + comorbidity and life style factors. ICC, intraclass correlation *p < 0.05, **p < 0.001.

**TABLE 3:** Mixed linear regression of activity impact on cognition with covariates.

	Women				Men			

	Dementia-free		Dementia cases		Dementia-free		Dementia cases	
*z*-values of	β	CI 95%	β	CI 95%	β	CI 95%	β	CI 95%
**WT1**								
Inactive	Ref	(-)	Ref	(-)	Ref	(-)	Ref	(-)
Active	0.04	(−0.01–0.09)	0.07	(−0.07–0.21)	0.07**	(0.02–0.12)	0.02	(−0.15–0.19)
Very active	0.04	(−0.03–0.12)	0.19	(−0.07–0.45)	0.10**	(0.04–0.16)	−0.10	(−0.33–0.13)
ICC	0.37	⋅⋅	0.26	⋅⋅	0.391	⋅⋅	0.287	⋅⋅
***WT2***								
Inactive	Ref	(-)	Ref	(-)	Ref	(-)	Ref	(-)
Active	0.10***	(0.05–0.15)	0.03	(−0.17–0.24)	0.10***	(0.05–0.16)	0.15	(−0.08–0.38)
Very active	0.07	(−0.00–0.14)	0.08	(−0.29–0.46)	0.11**	(0.04–0.18)	0.24	(−0.07–0.55)
ICC	0.33	⋅⋅	0.364	⋅⋅	0.288	⋅⋅	0.493	⋅⋅
***DSCT***								
Inactive	Ref	(-)	Ref	(-)	Ref	(-)	Ref	(-)
Active	0.17***	(0.13–0.21)	0.14*	(0.01–0.27)	0.12***	(0.08–0.16)	−0.00	(−0.14–0.13)
Very active	0.16***	(0.10–0.22)	0.37**	(0.14–0.60)	0.13***	(0.08–0.18)	0.22*	(0.03–0.41)
ICC	0.574	⋅⋅	0.363	⋅⋅	0.652	⋅⋅	0.395	⋅⋅
***MMSE***								
Inactive	Ref	(-)	Ref	(-)	Ref	(-)	Ref	(-)
Active	0.01	(−0.06–0.09)	0.13	(−0.45–0.71)	0.09**	(0.02–0.16)	0.49	(−0.37–1⋅34)
Very active	−0.03	(−0.12–0.07)	0.28	(−0.57–1⋅14)	0.08*	(0?00–0.16)	0.69	(−0.27–1⋅64)
ICC	0.335	⋅⋅	0.255	⋅⋅	0.38	⋅⋅	0.188	⋅⋅
***FTT***								
Inactive	Ref	(-)	Ref	(-)	Ref	(-)	Ref	(-)
Active	0.12***	(0.08–0.17)	−0.12	(−0.28–0.03)	0.08**	(0.03–0.13)	−0.04	(−0.22–0.13)
Very active	0.21***	(0.14–0.27)	−0.01	(−0.29–0.27)	0.10***	(0.04–0.16)	−0.02	(−0.26–0.22)
ICC	0.61	⋅⋅	0.397	⋅⋅	0.591	⋅⋅	0.61	⋅⋅
***Global CF***								
Inactive	Ref	(-)	Ref	(-)	Ref	(-)	Ref	(-)
Active	0.12***	(0.08–0.15)	−0.01	(−0.12–0.10)	0.10***	(0.07–0.13)	0.02	(−0.10–0.13)
Very active	0.11***	(0.07–0.16)	0.17	(−0.03–0.37)	0.11***	(0.07–0.15)	0.05	(−0.11–0.22)
ICC	0.495	⋅⋅	0.379	⋅⋅	0.464	⋅⋅	0.453	⋅⋅

Multiple mixed linear regression with nested id and z-values of the five cognitive tests and global cognitive test score as outcome. All models are adjusted for age, time, and education. β is the β-coefficient for active and very active, with inactive as reference. ICC, intraclass correlation. *p < 0.05, **p < 0.01, ***p < 0.001.

Later born dementia-free women remember on average 0.8 and 1 word more in Word test 1 and Word test 2, than the later born (Supplementary Appendix Table 5). For men, these numbers are a little lower (0.6 and 0.9). Other tests have even more improvement, and the sex difference is significant. How these improvements play out in everyday life for participants is not an aim of this study, and therefore not tested, but it shows promise.

## Discussion

Leisure time PA was positively associated with global cognition only in those remaining dementia-free, and the results were indifferent to the choice of cognitive measurement. There was an increasing gap in cognitive scores with age between the active and inactive in the dementia-free group, a phenomenon not present in the group developing dementia. For them, cognitive scores progressed similarly with age across LTPA groups.

For women who later developed dementia, we found a different association with LTPA in Digit Symbol Coding Test. This is the test most sensitive to cognitive change, and it applies to working memory, processing speed, visuospatial processing, and attention (Wechsler, 2008; Jaeger, 2018). For women in the dementia group, these scores improved significantly for those who were active compared to those who were inactive. Only very active men had an association with LTPA on this cognitive test. Short-term memory also was improved in women developing dementia, but after adjusting for education, this association disappeared. This suggests that education is a strong mediator, as well as in people in a preclinical phase of dementia. An association with short-term memory was not present in men.

Our findings are comparable to findings in a prospective 5-year trial, where they used a different set of cognitive tests but tested the same cognitive domains as in our study (Sokołowski et al., 2021). High and moderate intensive training programmes had no effect on cognition in a dementia-free population, but there was a positive effect from high cardiorespiratory fitness at baseline. Our participants reporting high LTPA are accordingly likely to be more fit and have higher cardiorespiratory fitness. Thus, the positive effect on cognition may be driven through the same pathways in the brain, such as brain-derived neurotrophic factor. A decrease in brain-derived neurotrophic factor has been found in people with neurodegenerative diseases, and exercise has been shown to increase these levels in the hippocampus, promoting learning and memory (El Hayek et al., 2019). It has even been associated with a 2% increase in hippocampal volume (Erickson et al., 2011). Furthermore, an American umbrella review found some evidence of a larger effect of exercise in preventing dementia when the study samples included a higher percentage of women (Erickson et al., 2019). This is supported by our study, finding an effect of LTPA on some cognitive areas, even in women developing dementia. In men in the preclinical phase of dementia, LTPA had a weaker effect on these cognitive domains. This could be due to the highly sensitive qualities of the Digit Symbol Coding Test (Jaeger, 2018). For short-term memory, data from a large prospective cohort showed that women had better memory scores than men, and memory decline was faster for men than for women (Bloomberg et al., 2021). This was confirmed by our study, which showed a positive association between short-term memory and activity for both sexes. The novel finding in our study was that this association remained in women who later developed dementia. However, when adjusted for education, the relationship no longer reached statistical significance, suggesting that the effect of education makes the sex differences lesser.

A study in the United Kingdom has found sex and birth cohort differences. Men born earlier in the same cohort had better fluency scores than women, but the effect was reversed in later-born cohorts (Bloomberg et al., 2021). They argued that secular changes in education levels differed between sexes, and this was the basis for better performance in later-born women. Participants in our Norwegian study endured the same changes in education levels over the last decade. High education is known to be associated with higher cognitive performance, and it has been suggested to be the largest contributor to cognitive reserve (Stern, 2009). In addition, findings from a recent study suggested that physical activity was a modulator of cognitive reserve, explaining the discrepancy between the degree of cognitive impairment and brain imaging abnormalities in a group of older participants (average age of 81 years) (Sato et al., 2021). They tested several known risk factors for dementia, but only found leisure time activity and education to contribute to the cognitive reserve. The cognitive reserve theory (Stern, 2009) postulates that those with a high cognitive reserve do not escape dementia; their symptoms debut later and are more severe. This shows as a preventive effect in statistical analyses, and it could explain some of the overrepresentation of dementia in women, as our oldest women today have less education, and so lower cognitive reserve, than men born at the same time, concurrently outliving them. However, it is also possible that physical activity has a greater effect on the female brain, and that the Word test 1 and the Digit Symbol Coding Test are the only tests sensitive enough to capture it.

The MMSE results deviated from the ones of the other four tests. This could be due to lower n and shorter follow-up time, as it was performed only in Tromsø6 and Tromsø7. In addition, the MMSE has been suggested to be less sensitive in cognitively healthy people, as it probably has a ceiling and floor effect (Philipps et al., 2014). This might explain the findings in our presumably dementia-free or pre-onset dementia population.

Our study found that subjects with the same level of education, sex, and age had lower cognitive scores before the onset of dementia compared to the dementia-free subjects. Our findings are similar to those from the Whitehall Study, where accelerating cognitive decline was observed 8–10 years before the onset of dementia (Sabia et al., 2017b).

The cognition-modulating impact of physical activity in healthy brains does not seem to have the same effect in the brains of males who later developed dementia as it does in female brains with subclinical dementia pathology.

However, compared to the inactive, those who have dementia-free brains and are active show increasing improvement in cognitive test scores with age. As a later dementia diagnosis cannot be predicted, health authorities should encourage physical activity to promote cognitive health in adults.

### Strengths and Limitations

#### Strengths

This large population-based study with a wide age span and long follow-up generated over 1,000 dementia cases. Cognitive tests were performed up to 19 years before the onset of dementia, and information about baseline risk factors was available. The measurements and assessments were performed in a standardized manner.

#### Limitations

Physical activity was self-reported at baseline, but it showed good agreement with accelerometer-assessed activity (Morseth and Hopstock, 2020). The Saltin–Grimby scale does not allow to do dose-effect measurements, nor does it distinguish between different types of physical activity. The MMSE did not have good consistency with the other tests. Unfortunately, we did not have good measurements of hearing to include in this study.

The study included only dementia patients whose diagnoses were registered in hospital records. Furthermore, only few additional dementia cases were identified from death certificates, and it is possible that undetected later dementia in the non-case group would weaken our findings. We did not have access to the participants’ APOE e4 status, but we did not analyze dementia subtypes either.

## Conclusion

Physical Activity has a positive association with global cognition function only in healthy brains. However, if already in the preclinical phase of dementia, PA does not improve overall cognition. In the dementia-free, the gap between active and inactive increases with increasing age, favoring active dementia-free people. PA impacts cognitive domains in men and women differently, with a larger effect on women.

## Data Availability Statement

Data cannot be made public as legal restrictions are set by the Tromsø Study Data and Publication Committee to control data and prevent potential reverse identification of de-identified sensitive participant information. The data can be made available from the Tromsø Study for researchers at the Tromsø Study Data and Publication Committee. Contact information: The Tromsø Study, Department of Community Medicine, Faculty of Health Sciences, UiT The Arctic University of Norway; e-mail: tromsous@uit.no.

## Ethics Statement

The studies involving human participants were reviewed and approved by the Regional Committee for Medical and Health Research Ethics (REK Helse Sør-Øst 2016/389). All participants consented to the retrieval of medical events or death from hospital records and health registries.

## Author Contributions

BJ: data curation, formal analysis, investigation, methodology, analysis tools, visualization, and writing – original draft. BS: funding acquisition, methodology, and writing – review and editing. GL: formal analysis, methodology, and writing – review and editing. IM: project administration, supervision, and rewriting – review and editing. HS: data curation, funding acquisition, methodology, project administration, analysis tools, supervision, and writing – review and editing. All authors contributed to the article and approved the submitted version.

## Conflict of Interest

The authors declare that the research was conducted in the absence of any commercial or financial relationships that could be construed as a potential conflict of interest.

## Publisher’s Note

All claims expressed in this article are solely those of the authors and do not necessarily represent those of their affiliated organizations, or those of the publisher, the editors and the reviewers. Any product that may be evaluated in this article, or claim that may be made by its manufacturer, is not guaranteed or endorsed by the publisher.
