# Supplementary material for: Leisure Time Physical Activities’ Association With Cognition and Dementia: A 19 Years’ Life Course Study
Source: Front Aging Neurosci. 2022 Jun 15;14:906678. doi: 10.3389/fnagi.2022.906678 (PMC9241436; doi:10.3389/fnagi.2022.906678)
Supplement: Supplementary file 1 [file Table_1.docx]

| SGPALS | PA |
| --- | --- |
| 1 | 0 |
| 2 | 1 |
| 3 | 2 |
| 4 | 2 |
| Physical activity light | |
| 1 | 0 |
| 2 | 0 |
| 3 | 0 |
| 4 | 0 |
| Physical activity hard | |
| 1 | 0 |
| 2 | 1 |
| 3 | 1 |
| 4 | 2 |

Table: Recoding of the different questionnaires from The Tromsø Study, into one ordinal variable, PA

Table e-1: Coding of the PA variable
