# Supplementary material for: Leisure Time Physical Activities’ Association With Cognition and Dementia: A 19 Years’ Life Course Study
Source: Front Aging Neurosci. 2022 Jun 15;14:906678. doi: 10.3389/fnagi.2022.906678 (PMC9241436; doi:10.3389/fnagi.2022.906678)
Supplement: Supplementary file 2 [file Table_2.docx]

Table e-2: Mixed linear regression of activity impact on cognition with covariates, without education.

|  | ***Women*** | | | | ***Men*** | | | |
| --- | --- | --- | --- | --- | --- | --- | --- | --- |
|  | ***Dementia-free*** | | ***Dementia cases*** | | ***Dementia-free*** | | ***Dementia cases*** | |
| ***z-values of*** | ***β*** | ***CI 95%*** | ***β*** | ***CI 95%*** | ***β*** | ***CI 95%*** | ***β*** | ***CI 95%*** |
| ***WT1*** |  |  |  |  |  |  |  |  |
| Inactive | Ref | (-) | Ref | (-) | Ref | (-) | Ref | (-) |
| Active | 0.08^**^ | (0.03- 0.13) | 0.12 | (-0.02- 0.26) | 0.10^***^ | (0.05- 0.16) | 0.02 | (-0.15- 0.19) |
| Very active | 0.12^***^ | (0.05- 0.19) | 0.26^*^ | (0.01- 0.52) | 0.16^***^ | (0.09- 0.22) | -0.06 | (-0.29- 0.17) |
| ***WT2*** |  |  |  |  |  |  |  |  |
| Inactive | Ref | (-) | Ref | (-) | Ref | (-) | Ref | (-) |
| Active | 0.14^***^ | (0.09- 0.19) | 0.09 | (-0.11- 0.30) | 0.13^***^ | (0.07- 0.19) | 0.15 | (-0.07- 0.38) |
| Very active | 0.14^***^ | (0.06- 0.21) | 0.23 | (-0.15- 0.61) | 0.15^***^ | (0.09- 0.22) | 0.26 | (-0.05- 0.57) |
| ***DSCT*** |  |  |  |  |  |  |  |  |
| Inactive | Ref | (-) | Ref | (-) | Ref | (-) | Ref | (-) |
| Active | 0.20^***^ | (0.16- 0.25) | 0.19^**^ | (0.07- 0.32) | 0.15^***^ | (0.11- 0.20) | 0.03 | (-0.11- 0.18) |
| Very active | 0.22^***^ | (0.16- 0.28) | 0.46^***^ | (0.23- 0.69) | 0.18^***^ | (0.13- 0.23) | 0.30^**^ | (0.11- 0.49) |
| ***MMSE*** |  |  |  |  |  |  |  |  |
| Inactive | Ref | (-) | Ref | (-) | Ref | (-) | Ref | (-) |
| Active | 0.05 | (-0.03- 0.12) | 0.18 | (-0.40- 0.75) | 0.12^**^ | (0.05- 0.19) | 0.41 | (-0.46- 1.28) |
| Very active | 0.05 | (-0.05- 0.14) | 0.36 | (-0.48- 1.21) | 0.13^**^ | (0.05- 0.21) | 0.68 | (-0.29- 1.66) |
| ***FTT*** |  |  |  |  |  |  |  |  |
| Inactive | Ref | (-) | Ref | (-) | Ref | (-) | Ref | (-) |
| Active | 0.14^***^ | (0.10- 0.19) | -0.08 | (-0.23- 0.08) | 0.09^***^ | (0.05- 0.14) | -0.03 | (-0.20- 0.14) |
| Very active | 0.25^***^ | (0.19- 0.31) | 0.10 | (-0.19- 0.38) | 0.13^***^ | (0.08- 0.19) | -0.02 | (-0.25- 0.22) |
| ***Global CF*** |  |  |  |  |  |  |  |  |
| Inactive | Ref | (-) | Ref | (-) | Ref | (-) | Ref | (-) |
| Active | 0.15^***^ | (0.12- 0.18) | 0.04 | (-0.07- 0.15) | 0.12^***^ | (0.09- 0.16) | 0.03 | (-0.09- 0.14) |
| Very active | 0.18^***^ | (0.13- 0.22) | 0.26^*^ | (0.06- 0.46) | 0.15^***^ | (0.11- 0.20) | 0.09 | (-0.07- 0.25) |

*Table e-2: Multiple mixed linear regression with nested id and z-values of the five cognitive tests and global cognitive test score as outcome. All models are adjusted for age and time. β is the β-coefficient for active and very active, with inactive as reference. ICC, intraclass correlation. *p < 0.05, **p < 0.01, ***p < 0.001.*
