# Supplementary material for: Leisure Time Physical Activities’ Association With Cognition and Dementia: A 19 Years’ Life Course Study
Source: Front Aging Neurosci. 2022 Jun 15;14:906678. doi: 10.3389/fnagi.2022.906678 (PMC9241436; doi:10.3389/fnagi.2022.906678)
Supplement: Supplementary file 3 [file Table_3.docx]

Table e-3: Fixed effects of global cognitive score

|  | ***Women*** | | | | ***Men*** | | | |
| --- | --- | --- | --- | --- | --- | --- | --- | --- |
| ***Z-values*** | ***Dementia-free*** | | ***Dementia cases*** | | ***Dementia-free*** | | ***Dementia cases*** | |
| ***Global CF*** | ***β*** | ***CI 95%*** | ***β*** | ***CI 95%*** | ***β*** | ***CI 95%*** | ***β*** | ***CI 95%*** |
| ***Model 1*** |  |  |  |  |  |  |  |  |
| Inactive | Ref | (-) | Ref | (-) | Ref | (-) | Ref | (-) |
| Active | 0.20^***^ | (0.16- 0.23) | 0.07 | (-0.04- 0.18) | 0.16^***^ | (0.12- 0.19) | 0.07 | (-0.05- 0.20) |
| Very active | 0.22^***^ | (0.17- 0.27) | 0.31^**^ | (0.11- 0.52) | 0.20^***^ | (0.16- 0.24) | 0.16 | (-0.01- 0.33) |
| ***Model 2*** |  |  |  |  |  |  |  |  |
| Inactive | Ref | (-) | Ref | (-) | Ref | (-) | Ref | (-) |
| Active | 0.15^***^ | (0.11- 0.18) | 0.01 | (-0.10- 0.12) | 0.12^***^ | (0.09- 0.16) | 0.06 | (-0.06- 0.18) |
| Very active | 0.13^***^ | (0.09- 0.18) | 0.19 | (-0.01- 0.40) | 0.14^***^ | (0.10- 0.18) | 0.10 | (-0.07- 0.27) |
| ***Model 3*** |  |  |  |  |  |  |  |  |
| Inactive | Ref | (-) | Ref | (-) | Ref | (-) | Ref | (-) |
| Active | 0.12^***^ | (0.08- 0.15) | -0.00 | (-0.11- 0.11) | 0.09^***^ | (0.05- 0.12) | 0.07 | (-0.05- 0.19) |
| Very active | 0.10^***^ | (0.05- 0.15) | 0.18 | (-0.02- 0.39) | 0.10^***^ | (0.06- 0.14) | 0.11 | (-0.06- 0.28) |
| ***Model 4*** |  |  |  |  |  |  |  |  |
| Inactive | Ref | (-) | Ref | (-) | Ref | (-) | Ref | (-) |
| Active | 0.07^***^ | (0.04- 0.11) | -0.04 | (-0.16- 0.09) | 0.06^***^ | (0.03- 0.10) | 0.05 | (-0.08- 0.18) |
| Very active | 0.06^*^ | (0.01- 0.11) | 0.16 | (-0.06- 0.38) | 0.06^**^ | (0.01- 0.10) | 0.05 | (-0.13- 0.23) |

Table e-3: Fixed effect of table 2. Multiple mixed linear regression for fixed effect with global cognitive test score as outcome. Model 1: adjusted for time and age, Model 2: Model 1 + education, Model 3: Model 2 + birth cohort, Model 4: Model 3 + comorbidity and life style factors. ^*^ p < 0.05, ^**^ p < 0.01, ^***^ p < 0.001
