# Supplementary material for: Leisure Time Physical Activities’ Association With Cognition and Dementia: A 19 Years’ Life Course Study
Source: Front Aging Neurosci. 2022 Jun 15;14:906678. doi: 10.3389/fnagi.2022.906678 (PMC9241436; doi:10.3389/fnagi.2022.906678)
Supplement: Supplementary file 4 [file Table_4.docx]

Table e-4: Fixed effects of separate cognitive tests

|  | ***Women*** | | | | ***Men*** | | | |
| --- | --- | --- | --- | --- | --- | --- | --- | --- |
|  | ***Dementia-free*** | | ***Dementia cases*** | | ***Dementia-free*** | | ***Dementia cases*** | |
| ***z-values of*** | ***β*** | ***CI 95%*** | ***β*** | ***CI 95%*** | ***β*** | ***CI 95%*** | ***β*** | ***CI 95%*** |
| ***WT1*** |  |  |  |  |  |  |  |  |
| Inactive | Ref | (-) | Ref | (-) | Ref | (-) | Ref | (-) |
| Active | 0.06^*^ | (0.01- 0.11) | 0.10 | (-0.04- 0.24) | 0.09^**^ | (0.03- 0.14) | 0.04 | (-0.13- 0.21) |
| Very active | 0.07 | (-0.01- 0.14) | 0.22 | (-0.05- 0.48) | 0.13^***^ | (0.06- 0.19) | -0.07 | (-0.30- 0.16) |
| ***WT2*** |  |  |  |  |  |  |  |  |
| Inactive | Ref | (-) | Ref | (-) | Ref | (-) | Ref | (-) |
| Active | 0.10^***^ | (0.05- 0.16) | 0.09 | (-0.12- 0.29) | 0.10^***^ | (0.05- 0.16) | 0.18 | (-0.06- 0.41) |
| Very active | 0.07 | (-0.01- 0.14) | 0.14 | (-0.24- 0.52) | 0.10^**^ | (0.04- 0.17) | 0.27 | (-0.05- 0.59) |
| ***DSCT*** |  |  |  |  |  |  |  |  |
| Inactive | Ref | (-) | Ref | (-) | Ref | (-) | Ref | (-) |
| Active | 0.20^***^ | (0.16- 0.25) | 0.15^*^ | (0.02- 0.28) | 0.18^***^ | (0.13- 0.23) | 0.03 | (-0.11- 0.17) |
| Very active | 0.17^***^ | (0.11- 0.24) | 0.39^**^ | (0.15- 0.62) | 0.20^***^ | (0.15- 0.26) | 0.19^*^ | (0.00- 0.38) |
| ***MMSE*** |  |  |  |  |  |  |  |  |
| Inactive | Ref | (-) | Ref | (-) | Ref | (-) | Ref | (-) |
| Active | 0.02 | (-0.05- 0.09) | 0.11 | (-0.47- 0.69) | 0.10^**^ | (0.03- 0.17) | 0.48 | (-0.37- 1.33) |
| Very active | -0.03 | (-0.12- 0.06) | 0.26 | (-0.60- 1.11) | 0.07 | (-0.00- 0.15) | 0.68 | (-0.27- 1.63) |
| ***FTT*** |  |  |  |  |  |  |  |  |
| Inactive | Ref | (-) | Ref | (-) | Ref | (-) | Ref | (-) |
| Active | 0.19^***^ | (0.14- 0.23) | -0.10 | (-0.25- 0.06) | 0.11^***^ | (0.06- 0.16) | -0.03 | (-0.22- 0.15) |
| Very active | 0.27^***^ | (0.20- 0.34) | 0.03 | (-0.25- 0.32) | 0.15^***^ | (0.09- 0.21) | 0.07 | (-0.19- 0.32) |

Table e-4: Multiple mixed linear regression with fixed effects of z-values of the 5 cognitive tests and global cognitive tests as outcome. All models are adjusted for age, time and education. β is the β-coefficient for active and very active, with inactive as reference. ICC: Intra class correlation. ^*^ p < 0.05, ^**^ p < 0.01, ^***^ p < 0.001
