# Supplementary material for: Leisure Time Physical Activities’ Association With Cognition and Dementia: A 19 Years’ Life Course Study
Source: Front Aging Neurosci. 2022 Jun 15;14:906678. doi: 10.3389/fnagi.2022.906678 (PMC9241436; doi:10.3389/fnagi.2022.906678)
Supplement: Supplementary file 5 [file Table_5.docx]

Table e-5

Table e-5: Mean unstandardized score cognitive tests with standard deviation.

|  | Women Dementia-free | | | | Women Dementia cases | | | |
| --- | --- | --- | --- | --- | --- | --- | --- | --- |
|  | Tromsø5 | Tromsø6 | Tromsø7 | P-value | Tromsø5 | Tromsø6 | Tromsø7 | P-value |
|  | N=2,541 | N=3,777 | N=4,054 |  | N=605 | N=371 | N=109 |  |
| Word Test 1 | 6.5 (1.9) | 6.8 (1.9) | 7.3 (2.0) | <0.001 | 5.6 (1.9) | 5.1 (2.0) | 4.5 (2.0) | <0.001 |
| Word Test 2 | 21.3 (2.5) | 22.1 (1.9) | 22.3 (1.8) | <0.001 | 20.0 (2.9) | 20.3 (2.9) | 20.0 (2.6) | 0.52 |
| Digit Symbol Coding Test | 31.2 (14.5) | 40.6 (13.2) | 42.9 (11.5) | <0.001 | 23.3 (12.8) | 26.3 (11.4) | 25.8 (11.6) | 0.006 |
| MMSE | N/A | 28.3 (1.6) | 27.9 (2.3) | <0.001 | N/A | 27.0 (2.6) | 24.7 (4.2) | <0.001 |
| Finger Tapping Test | 46.7 (10.0) | 47.6 (10.2) | 50.8 (8.6) | <0.001 | 44.1 (10.5) | 39.9 (12.1) | 45.0 (8.8) | <0.001 |
|  |  |  |  |  |  |  |  |  |
|  | **Men Dementia-free** | | | | **Men Dementia cases** | | | |
|  | Tromsø5 | Tromsø6 | Tromsø7 | P-value | Tromsø5 | Tromsø6 | Tromsø7 | P-value |
|  | N=1,927 | N=3,082 | N=3,395 |  | N=425 | N=247 | N=77 |  |
| Word Test 1 | 6.1 (1.9) | 6.4 (1.9) | 6.8 (1.9) | <0.001 | 5.2 (2.0) | 4.5 (1.9) | 3.9 (1.6) | <0.001 |
| Word Test 2 | 21.1 (2.4) | 21.7 (2.0) | 22.0 (1.8) | <0.001 | 20.1 (2.7) | 20.5 (2.6) | 19.6 (2.7) | 0.12 |
| Digit Symbol Coding Test | 29.9 (13.8) | 37.4 (12.3) | 39.0 (10.9) | <0.001 | 22.7 (12.1) | 24.9 (12.2) | 24.1 (9.6) | 0.15 |
| MMSE | N/A | 28.1 (1.6) | 27.7 (1.9) | <0.001 | N/A | 26.6 (2.6) | 23.6 (5.7) | <0.001 |
| Finger Tapping Test | 51.8 (9.8) | 53.1 (10.1) | 55.0 (8.5) | <0.001 | 48.5 (10.6) | 45.2 (11.6) | 48.0 (9.0) | 0.007 |

Table e-5: Mean cognitive test score for the five tests stratified on survey, sex and endpoint. All numbers are mean with standard deviation in parentheses. P-values are obtained by ANOVA test. MMSE: Mini Mental Status Evaluation.
