# Supplementary material for: Leisure Time Physical Activities’ Association With Cognition and Dementia: A 19 Years’ Life Course Study
Source: Front Aging Neurosci. 2022 Jun 15;14:906678. doi: 10.3389/fnagi.2022.906678 (PMC9241436; doi:10.3389/fnagi.2022.906678)
Supplement: Supplementary file 6 [file Table_6.docx]

Table e-6

| ICD-codes | Diagnoses |
| --- | --- |
| G30.0 | Alzheimer disease with early onset |
| G30.1 | Alzheimer disease with late onset |
| G30.8 | Other Alzheimer disease |
| G30.9 | Alzheimer disease, unspecified |
| G31.8 | Other specified degenerative diseases of nervous system including LBD |
| F00.0 | Dementia in Alzheimer disease with early onset |
| F00.1 | Dementia in Alzheimer disease with late onset |
| F00.2 | Dementia in Alzheimer disease, atypical or mixed type |
| F00.9 | Dementia in Alzheimer disease, unspecified |
| F01.0 | Vascular dementia |
| F01.1 | Multi-infarct dementia |
| F01.2 | Subcortical vascular dementia |
| F01.3. | Mixed cortical and subcortical vascular dementia |
| F01.8 | Other vascular dementia |
| F01.9 | Vascular dementia, unspecified |
| F02.0 | Dementia in Pick disease |
| F02.1 | Dementia in Creutzfeldt-Jakob disease |
| F02.2 | Dementia in Huntington disease |
| F02.3 | Dementia in Parkinson disease |
| F02.4 | Dementia in human immunodeficiency virus [HIV] disease |
| F02.8 | Dementia in other specified diseases classified elsewhere |
| F03 | Unspecified dementia |

Table e-6: All included ICD-codes in the register coding dementia diagnoses.
