# Supplementary figures and images for: Leisure Time Physical Activities’ Association With Cognition and Dementia: A 19 Years’ Life Course Study
Source: Front Aging Neurosci. 2022 Jun 15;14:906678. doi: 10.3389/fnagi.2022.906678 (PMC9241436; doi:10.3389/fnagi.2022.906678)

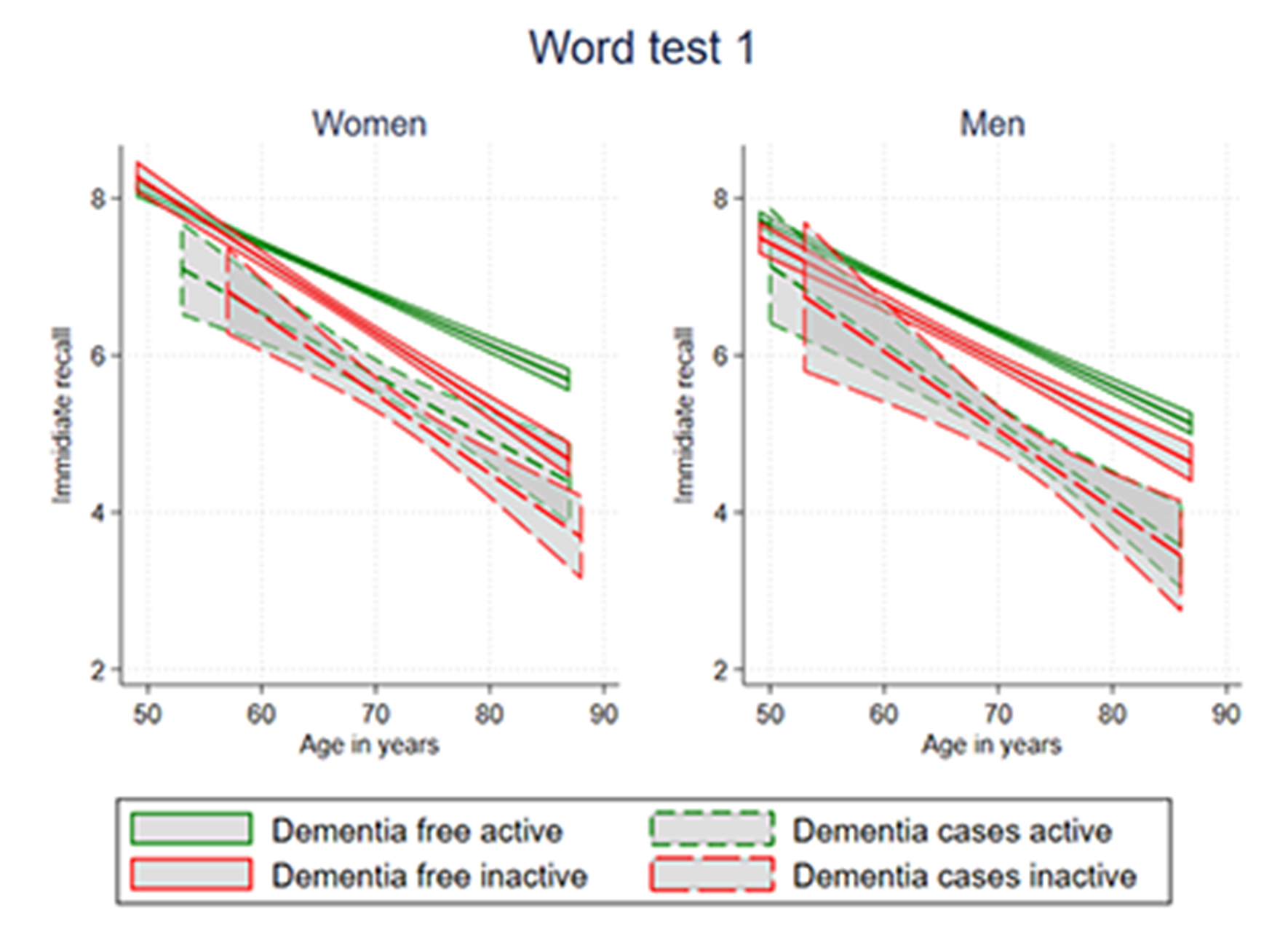

Supplement: Supplementary file 7 [file Image_1.tif]

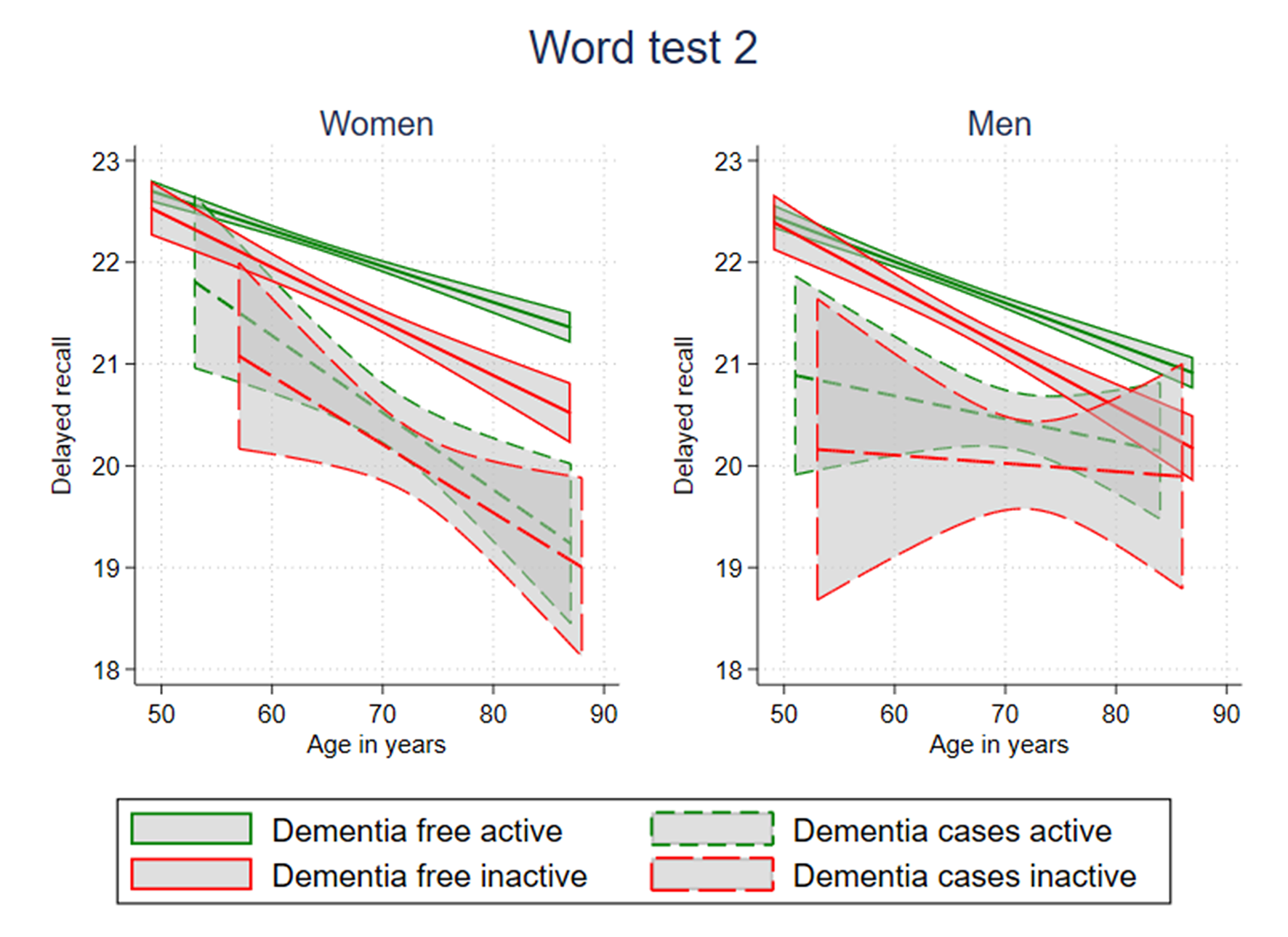

Supplement: Supplementary file 8 [file Image_2.tif]

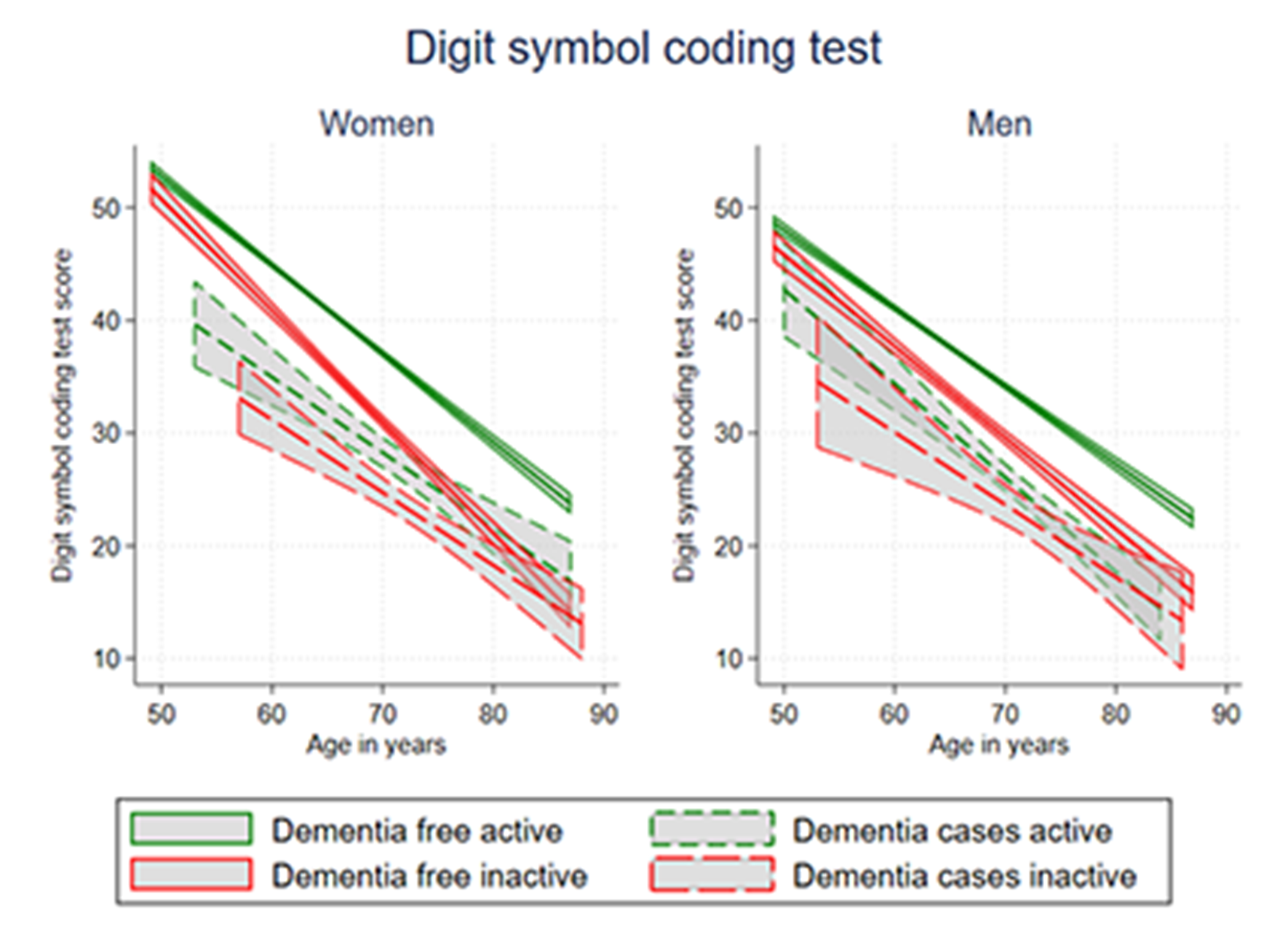

Supplement: Supplementary file 9 [file Image_3.tif]

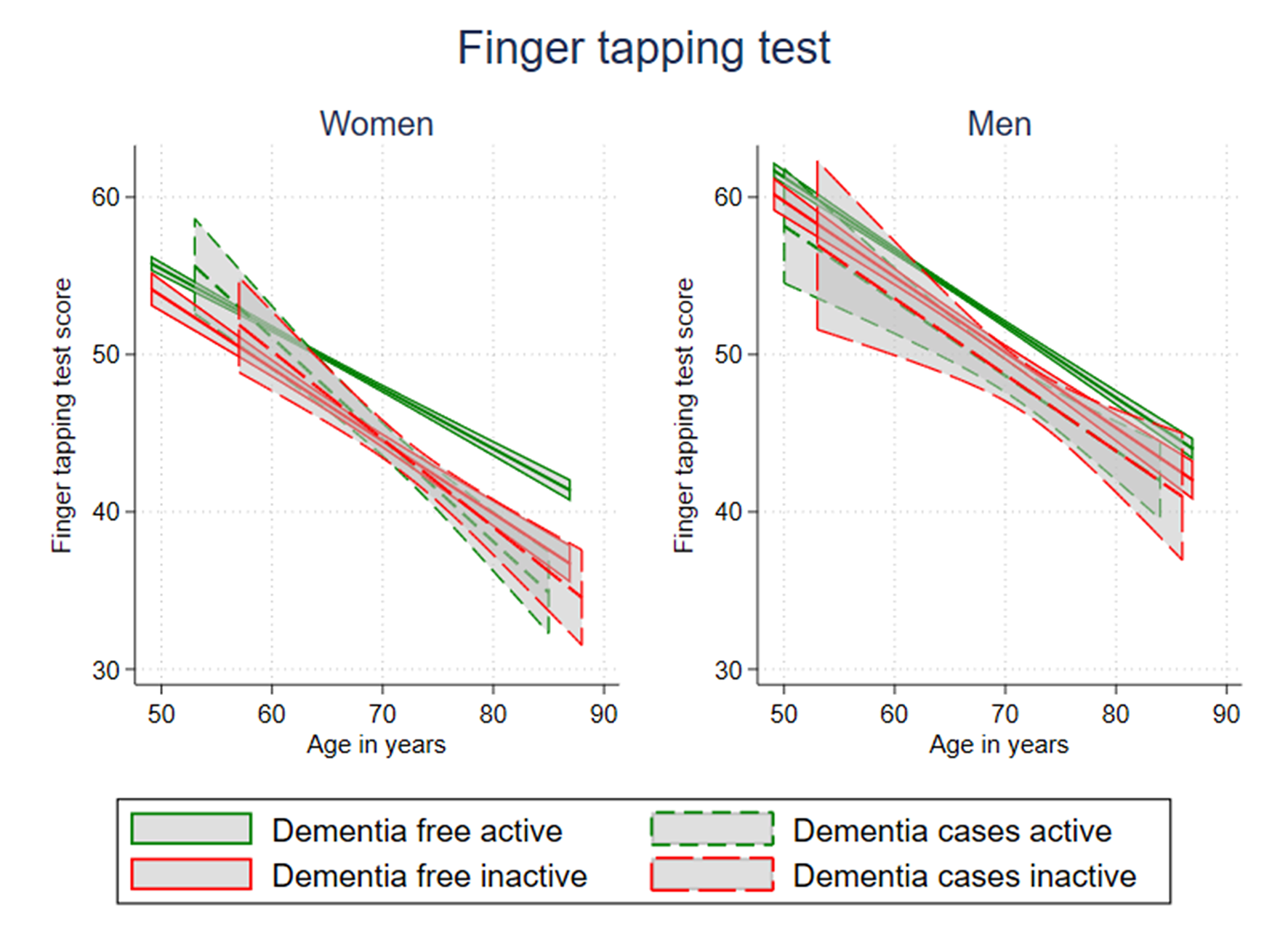

Supplement: Supplementary file 10 [file Image_4.tif]

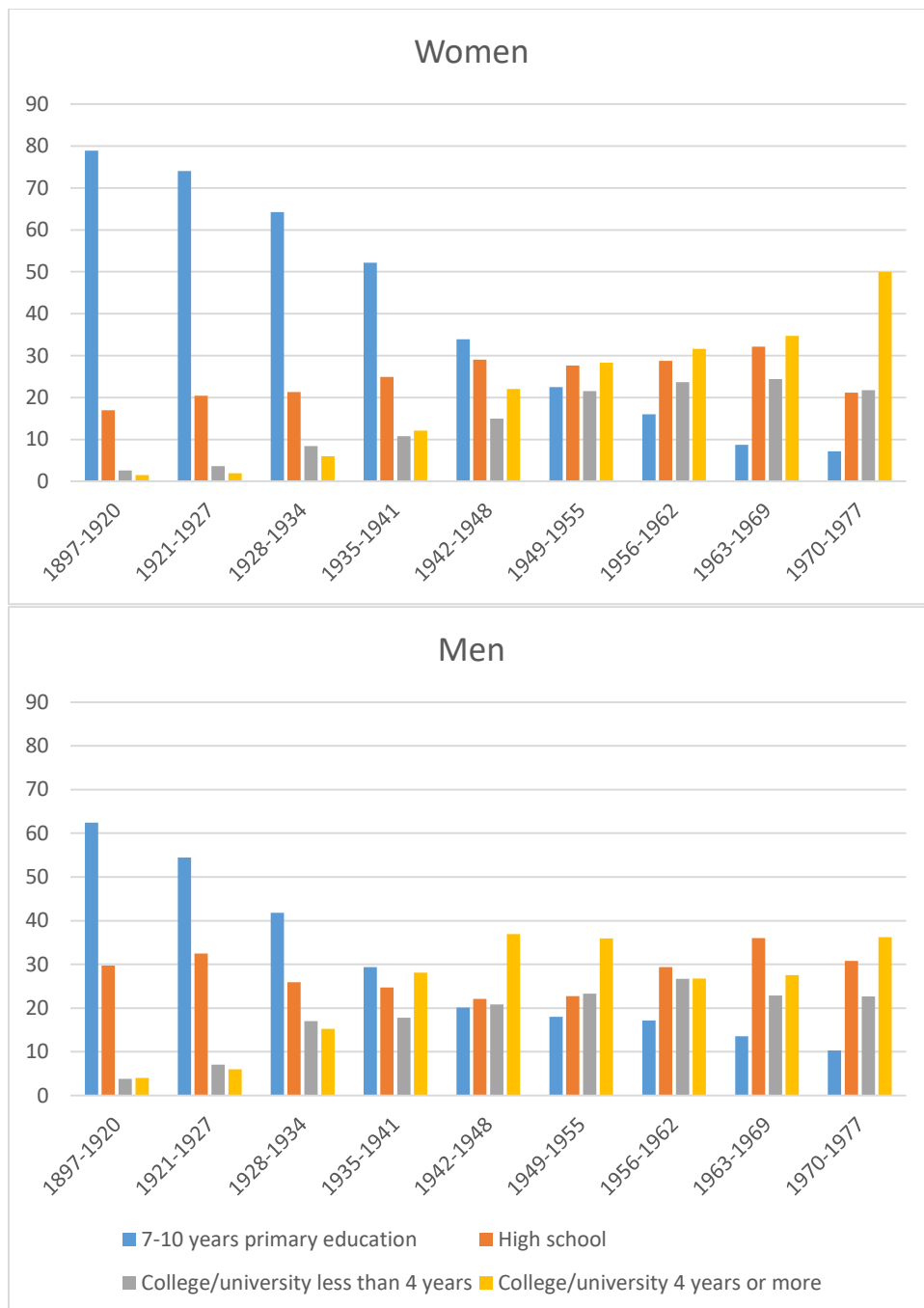

Figure e-5: Education over the last century

Supplement: Supplementary file 11 [file Image_5.pdf]
